# Chronic Liver Enzyme Elevation and Use of Contemporary ARVs Among People With HIV

**DOI:** 10.1093/ofid/ofae308

**Published:** 2024-06-08

**Authors:** Ashley O Roen, Lars Peters, Gilles Wandeler, Marc van der Valk, Robert Zangerle, Huldrych F Günthard, Ferdinand Wit, Cristina Mussini, Stéphane De Wit, Antonella d’Arminio Monforte, Jörg Janne Vehreschild, Antonella Castagna, Nadine Jaschinski, Vani Vannappagari, Linda Chen, Joan Tallada, John C’mar, Amanda Mocroft, Lene Ryom

**Affiliations:** Institute for Global Health, University College London, London, UK; CHIP, Rigshospitalet, University of Copenhagen, Copenhagen, Denmark; Department of Infectious Diseases, Bern University Hospital, University of Bern, Bern, Switzerland; Stichting HIV Monitoring Amsterdam, Amsterdam, The Netherlands; Amsterdam University Medical Centers, University of Amsterdam, Division of Infectious Diseases, and Amsterdam Institute for Infection and Immunity, Amsterdam, The Netherlands; Austrian HIV Cohort Study (AHIVCOS), Medizinische Universität Innsbruck, Innsbruck, Austria; Department of Infectious Diseases and Hospital Epidemiology, University Hospital Zurich, Zurich, Switzerland; Institute of Medical Virology, University of Zurich, Zurich, Switzerland; AIDS Therapy Evaluation in the Netherlands (ATHENA) Cohort, HIV Monitoring Foundation, Amsterdam, The Netherlands; Modena HIV Cohort, Università degli Studi di Modena, Modena, Italy; CHU Saint-Pierre, Centre de Recherche en Maladies Infectieuses a.s.b.l., Brussels, Belgium; Italian Cohort Naive Antiretrovirals (ICONA), ASST Santi Paolo e Carlo, Milano, Italy; University Hospital Cologne, Cologne, Germany; San Raffaele Scientific Institute, Università Vita-Salute San Raffaele, Milano, Italy; CHIP, Rigshospitalet, University of Copenhagen, Copenhagen, Denmark; ViiV Healthcare, Research Triangle Park, North Carolina, USA; Gilead Science, Foster City, California, USA; European AIDS Treatment Group, Brussels, Belgium; MSD, North Wales, Pennsylvania, USA; Institute for Global Health, University College London, London, UK; CHIP, Rigshospitalet, University of Copenhagen, Copenhagen, Denmark; CHIP, Rigshospitalet, University of Copenhagen, Copenhagen, Denmark; Department of Infectious Diseases 144, Hvidovre University Hospital, Copenhagen, Denmark; Department of Clinical Medicine, University of Copenhagen, Copenhagen, Denmark

**Keywords:** HIV, Chronic liver enzyme elevation (cLEE), HCV; ART

## Abstract

**Background:**

While use of some older antiretroviral drugs (ARVs) is associated with chronic liver enzyme elevation (cLEE), the impact of newer ARVs remains unknown.

**Methods:**

People with HIV enrolled in the RESPOND cohort who started an ARV after January 1, 2012 were included (baseline). The primary outcome was first cLEE individuals were censored at first of cLEE, last visit, death, or December 31, 2021. Incidence rates (IRs; events/1000 person-years) were calculated for each ARV overall and by ARV exposure (6–12 months, 1–2 years, and 2+ years). Poisson regression was used to estimate the incidence rate ratio (IRR) of cLEE and its association with individual ARVs and ARV class.

**Results:**

Of 17 106 individuals included contributing 87 924 person-years of follow-up, 1932 (11.3%) experienced cLEE (incidence rate [IR], 22.0; 95% CI, 21.0–23.0). There was no evidence of a cumulative ARV effect on cLEE incidence, (6–12 months: IR, 45.8; 95% CI, 41.4–50.19; 1–2 years: IR, 34.3; 95% CI, 31.5–37.4; and 2+ years: IR, 18.5; 95% CI, 17.4–19.7). Any use (vs no prior use) of non-nucleoside reverse transcriptase inhibitors (NNRTIs) as a class and tenofovir disoproxil fumarate (TDF) was independently associated with an increased IRR of cLEE, and any use of darunavir (DRV) was associated with a decreased risk of cLEE.

**Conclusions:**

cLEE is common and more frequent during the first year after initiating new ARVs. With a >5-year median follow-up, we found no short-term liver safety concerns with the use of INSTIs. Use of NNRTIs and TDF was associated with an increased cLEE risk, while DRV was associated with lower risk.

Although life expectancy has greatly increased for people with HIV, there remains a large difference in comorbidity-free years, where people with HIV mono-infection in the Western setting have an estimated 16.3 fewer healthy years [1] with a greater comorbidity burden [2] than HIV-negative individuals. Liver disease is common among people with HIV in Western settings, and although it has been declining in recent years, it accounts for 13%–18% of all-cause mortality and remains a leading cause of non-AIDS-related deaths [3–6]. This could be attributed to coinfection with viral hepatitis C (HCV) or hepatitis B (HBV), substance abuse, nodular regenerative hyperplasia, and nonalcoholic fatty liver disease [7, 8]. Poorly controlled HIV infection has also been shown to be an independent risk factor of liver fibrosis [9].

Chronic liver enzyme elevation (elevations in transaminases as a marker for hepatocyte turnover) is common in people with HIV with and without HCV/HBV coinfection [10–13], yet the clinical significance remains unclear due to lack of longer-term follow-up in many studies, particularly for newer antiretroviral drugs (ARVs) including integrase strand inhibitors (INSTIs). Most research on liver enzyme elevation in people with HIV has focused on acute liver injury, where liver enzyme elevation is elevated 3–5 times the upper limit of normal (ULN) [14–17]. Limited data are available on the risk of developing chronic moderate alanine aminotransferase (ALT) elevations among those prescribed newer ARVs with or without coinfections. Previous analyses from the large Data Collection on Adverse Events of Anti-HIV Drugs (D:A:D) Study reported that cumulative exposure to the older ARVs stavudine, didanosine, and amprenavir, and also to TDF, regardless of viral hepatitis status, were associated with chronic liver enzyme (transaminase) elevation (cLEE), end-stage liver disease (ESLD), and hepatocellular carcinoma [18], while other studies have found no such association [19, 20]. These studies were published during the beginning of the INSTIs era and did not investigate association with INSTIs. Data from the US military HIV Natural History Study (NHS) among 2779 military beneficiaries not coinfected with HCV/HBV suggest that INSTI-based regimens could be protective against cLEE [21].

Here we aim to identify risk factors associated with cLEE, focusing on commonly prescribed ARVs in RESPOND, namely INSTIs (dolutegravir [DTG], raltegravir [RAL], cobicistat boosted elvitegravir [EVG/c], and bictegravir [BIC]), protease inhibitors (PIs; boosted darunavir [DRV/b] and atazanavir [ATV/b]), non-nucleoside reverse transcriptase inhibitors (NNRTIs; rilpivirine [RPV] and efavirenz [EFV]), and backbone nucleoside reverse transcriptase inhibitors (NRTIs) that have been associated with liver impairment (tenofovir disoproxil fumarate [TDF]) and ALT normalization (tenofovir alafenamide [TAF]) [22].

## METHODS

### Data

The International Cohort Consortium of Infectious Diseases (RESPOND) is a cohort collaboration of ∼39 000 people with HIV among 19 cohorts from Europe and Australia, as described elsewhere [23]. Briefly, participating cohorts collect standardized HIV data from clinical visits, which are annually sent to the central coordinating center using the HIV Cohorts Data Exchange Protocol (HICDEP) where data are centrally validated. More information can be found at https://www.chip.dk/Studies/RESPOND. For this analysis and consistent with RESPOND guidelines for study inclusion, we included cohorts where ≥70% of the individuals under follow-up had ≥1 ALT measurement per year for the duration of the study period as these are cohorts with adequate data quality for our outcome of interest.

### Definitions

Baseline was defined as the date of initiation of a new ARV after January 1, 2012, to which individuals had not been previously exposed. As described above, the ARVs considered were INSTIs (DTG, RAL, EVG/c, BIC); PIs (DRV/b and ATV/b), NNRTIs (RPV and EFV), and backbone NRTIs (TDF and TAF). In an intention-to-treat approach, individuals were followed up until the first of cLEE (defined below), date of last visit, death, or December 31, 2022 (administrative censoring date).

Individuals were classified as having cLEE using the definition outlined by D:A:D in previous studies [24], namely by chronic ALT elevations greater than the ULN (males and females >50, >35 U/L, respectively) at ≥2 visits spanning at least 6 months and within 2 years, allowing for 1 normal value in between 2 elevated values. The first elevated ALT date after 6 months was used as the event date, as done previously [24].

Individuals were included if they had an HIV-RNA, CD4, and ALT measurement in the year preceding baseline and all ALT measurements in the year preceding baseline were normal (ALT < ULN), with at least 2 ALT measurements within 2 years of baseline.

### Statistical Analysis

Baseline characteristics of age, sex, ethnic origin, HIV transmission risk group, geographical region of care, calendar year, alcohol use, smoking status, ARV status (starting ART from a treatment-naïve or -experienced state), use of individual ARVs (as prespecified above), CD4 nadir, baseline HIV-RNA (taken in the year preceding baseline), HBV and HCV status, diabetes, body mass index (BMI), prior cLEE, and ESLD were described as numbers and percentages for categorical variables or medians and interquartile range (IQR) for continuous variables and stratified by whether the individual developed cLEE. Positive HCV status was defined by use of anti-HCV medication, a positive HCV antibody test, a positive HCV-RNA qualitative test, HCV-RNA >615 IU/mL, and/or a positive genotype test. Positive HBV status was defined by a positive HBV surface antigen test and/or HBV-DNA >357 IU/mL, and dyslipidemia was defined as random total cholesterol ≥240 mg/dL, high-density lipoprotein (HDL) <35 mg/dFl, triglyceride ≥200 mg/dL, or initiation of lipid-lowering therapy. Diabetes was defined as blood glucose levels >7 mmol/L (126 mg/dL) or HbA1C >6.5% (48 mmol/L) and use of antidiabetic treatment. ESLD was defined as clinical symptoms of end-stage liver failure in participants with chronic liver disease, based on the diagnosis documented in a clinical note of (a) endoscopically verified bleeding from gastric or esophageal varices; (b) hepatic encephalopathy stage III or IV; (c) hepatorenal syndrome; or (d) ascites; and a pathology report or fibro-scan report documenting advanced liver fibrosis or cirrhosis or liver transplantation. Sex/gender, ethnic origin/race, alcohol use and smoking status were self-reported.

Incidence rates (IRs; events/1000 person-years) and 95% CIs of cLEE were estimated for individual drugs and by main drug classes among the drugs in the prespecified list. Incidence rates were also estimated by time from initiating each drug of interest to ascertain if there was a cumulative association with longer drug exposure. We investigated the exposure categories 6–12 months, 1–2 years, and 2+ years (note: 0–6 months does not apply as per the definition of cLEE).

Poisson regression was used to estimate the incident rate ratio (IRR) of cLEE and its association with individual ARVs (compared with those not initiating the drug of interest), adjusting for prespecified key factors of baseline viral hepatitis status (positive, negative, or unknown), nadir CD4 at baseline (<350, 350–500, ≥500 cells/mm^3^), and HIV-RNA at baseline (<200, ≥200 copies/mL). Additional baseline risk factors for cLEE were univariably assessed, and those with *P* < .1 were included in multivariable models (final variable selection in results). Factors in multivariable models with *P* > .1 were omitted from the final multivariable model using backwards elimination. Variables removed were then one at a time included, and those with *P* < .1 were kept in the final model. Confounders investigated were age (continuous), sex/gender, ethnic origin/race (White, Black, other, unknown), HIV transmission risk group (men who have sex with men [MSM], injection drug users [IDUs], heterosexual contact, other, and unknown), geographical region (Western Europe + Australia, Southern Europe, Northern Europe, or Eastern Europe), calendar year (categorical), dyslipidemia (random total cholesterol >240 mg/dL, HDL <35 mg/dL, triglycerides >200 mg/dL, or initiation of lipid-lowering therapy), diabetes mellitus status, BMI (<25, ≥25, missing), and smoking status (current, prior, none, unknown). Robust standard errors were used to account for over- and underdispersion. In order to have adequate power (type I and II error rates of 0.05 and 0.2, respectively) to detect an IRR of ≥1.4 with 1932 events, we needed a follow-up ratio of 4:96 between the drug of interest and all other drugs as defined by Schoenfeld [25, 26].

We conducted multiple sensitivity analyses. First, we investigated an on-treatment approach, censoring at 90 days after any drug switch (to allow for a washout period). We also stratified the analysis by baseline viral hepatitis status, and we limited the analysis to those who were ART-naïve before baseline. To try and account for possible confounders for starting INSTIs, we adjusted for prior AIDS, tuberculosis (TB) diagnoses, and cardiovascular disease (CVD). And finally, any drugs we found to be associated with cLEE (NNRTIs [EFV, RPV] and TDF) were investigated individually among those not concomitantly prescribed the other drugs associated with cLEE (eg, EFV without concomitant prescriptions for RPV or TDF). This allowed us to see if the associations were truly independent or driven by 1 drug. DRV/b was examined without a concomitant prescription for TDF to examine if its protective effect was independent from TDF. All sensitivity analyses had fewer events than the main analysis, so only drugs with the power to detect an IRR of ≥1.4 were investigated, as in the main analysis described above.

Among those who developed cLEE, we defined severity per the AIDS Clinical Trial Group [27]: Below Grade 1: >ULN–1.25×ULN; Grade 1: 1.25–2.5×ULN; Grade 2: 2.5–5×ULN; Grade 3: 5–10×ULN or Grade 4 >10×ULN.

All analyses were performed using Stata/SE 18.0 from StataCorp LLC (College Station, TX, USA).

### Patient Consent

Participants consented to share data with RESPOND according to local requirements. Participants were pseudonymized at enrollment by assignment of a unique identifier by the participating cohort before data transfer to the main RESPOND database. All cohorts have approval to share data with RESPOND according to national and local requirements. Ethical approval was obtained, if required, from the relevant bodies for collection and sharing of data. Data are stored on secure servers at the RESPOND coordinating center in Copenhagen, Denmark, in accordance with current legislation and under approval by The Danish Data Protection Agency (approval number 2012-58-0004, RH-2018-15, January 26, 2018), under the EU General Data Protection Regulation (2016/679).

## RESULTS

Of 26 998 individuals in RESPOND among the 16 eligible cohorts who began a new ARV after 2012, we made the following exclusions. A total of 1521 were missing CD4 or HIV-RNA measurements in the year preceding baseline, 6140 had an abnormal ALT in the year preceding baseline, 1891 were missing an ALT measurement in the year preceding baseline, and 340 of the remaining individuals did not have ALT measurements taken after baseline (Supplementary Figure 1). Baseline characteristics of those included compared with those excluded were similar, apart from geographical region of care, where those included tended to be more likely to receive care in Western and Southern Europe and less likely to receive care in Northern and Eastern Europe (Supplementary Table 1).

A total of 17 106 individuals were included in the analysis, of whom 1932 (11.3%) went on to develop cLEE during 87 924 person-years of follow-up (PYFU), with a median follow-up (IQR) of 5.17 (2.97–7.22) per person, giving an IR of 22.0/1000 PYFU (95% CI, 21.0–23.0). Individuals included were mostly male (76%), White (71%), and MSM (49%) (Table 1). A little over 30% of individuals were ARV-naïve before baseline. The median follow-up for those who developed cLEE (IQR) was 2.2 (1.1–4.1) vs 5.5 (3.5–7.4) years in those without cLEE. Compared with those who did not develop cLEE during follow-up, those who developed cLEE were slightly more likely to be HCV- and/or HBV-positive at baseline (20.7% vs 18.5%), to have previously had cLEE at least 1 year before baseline (24% vs 14%), and had a slightly higher median ALT (IQR) at baseline (28 [22–35] vs 23 [17–30]).

**Table 1.** Baseline Characteristics of Individuals Who Did and Did Not Develop Chronic Liver Enzyme Elevation After Initiating a New ARV to Which They Have Not Been Exposed Previously

		Total		No cLEE		cLEE	
		No.	%	No.	%	No.	%
Gender	Male	13 018	76.1	11 576	76.3	1442	74.6
…	Female	4046	23.7	3560	25.2	486	25.2
…	Nonmale/female or unknown	42	0.2	38	0.2	4	0.2
HIV risk	MSM	8346	48.8	10 673	73.3	1417	73.3
…	IDU	1686	9.9	2192	10.2	198	10.2
…	Heterosexual	6019	35.2	662	6.1	118	6.1
…	Other	395	2.3	1647	10.3	199	10.3
…	Unknown	660	3.9	7416	48.1	930	48.1
Ethnic origin	White	12 090	70.7	1476	10.9	210	10.9
…	Black	2390	14	5334	35.5	685	35.5
…	Other	780	4.6	359	1.9	36	1.9
…	Unknown	1846	10.8	589	3.7	71	3.7
Geographical region	Western Europe	9534	55.7	8459	55.6	1075	55.6
…	Southern Europe	3664	21.4	3303	18.7	361	18.7
…	Northern Europe	1969	11.5	1724	12.7	245	12.7
…	Eastern Europe	1939	11.3	1688	13	251	13
ART starting year	2012/2013	3743	21.9	3104	33.1	639	33.1
…	2014/2015	4935	28.9	3497	32.5	627	32.5
…	2016/2017	4794	28.1	7680	33	636	33
…	2018/2019	2483	14.5	7909	29	559	29
…	2020/2021	995	5.8	4361	22.4	433	22.4
ART class	*INSTI*	9116	53.3	8185	53.9	931	48.2
…	DTG	5041	29.5	4552	30	489	25.3
…	EVG/c	1747	10.2	1557	10.3	190	9.8
…	RAL	1437	8.4	1223	8.1	214	11.1
…	*NNRTI*	3504	20.5	3021	19.9	483	25
…	EFV	882	5.2	735	4.8	147	7.6
…	RPV	2623	15.3	2287	15.1	336	17.4
…	*NRTI*	8369	48.9	7470	49.2	899	46.5
…	TAF	4287	25.1	3977	26.2	310	16
…	TDF	4184	24.5	3585	23.6	599	31
…	*PI*	3191	18.7	2783	18.3	408	21.1
…	DRV/b	2623	15.3	2307	15.2	316	16.4
…	ATV	569	3.3	477	3.1	92	4.8
ART-naive^a^	…	5271	30.8	4672	30.8	599	31
HCV/HBV-positive^a^	…	3201	18.7	2802	18.5	399	20.7
HCV-positive^a^	…	2665	15.6	2330	15.4	335	17.3
HBV-positive^a^	…	678	4	593	3.9	85	4.4
Prior cLEE^a^	…	2544	14.9	2081	13.7	463	24
Diabetes diagnosis before start^a^		641	3.7	555	3.7	86	4.5
ESLD diagnosis before start^a^		73	0.4	64	0.4	9	0.5
…	…	Median	IQR	Median	IQR	Median	IQR
Age^a^	…	47	(38–55)	47	(38–55)	45	(37–53)
CD4 nadir^a^	…	235	(113–362)	236	(114–363)	227	(109–356)
Baseline RNA^a^	…	1.6	(1.3–4.1)	1.6	(1.3–3.9)	1.6	(1.3–4.2)
Follow-up years	…	5.17	(2.97–7.22)	5.5	(3.5–7.4)	2.20	(1.14–4.07)
ALT^a^	…	23	(18–30)	23	(17–30)	28	(22–35)

Western Europe: Austria, Belgium, France, Germany, Luxembourg, Switzerland. Southern Europe: Argentina, Greece, Israel, Italy, Portugal, Spain. Northern Europe: Denmark, Finland, Iceland, Ireland, Australia, Netherlands, Norway, Sweden, United Kingdom. Eastern Europe: Albania, Belarus, Estonia, Georgia, Latvia, Lithuania, Russia, Ukraine, North Macedonia, Bosnia-Herzegovina, Croatia, Czech Republic, Hungary, Poland, Romania, Serbia, Slovenia, Slovakia.

Abbreviations: ALT, alanine transaminase; ATV, atazanavir; cLEE, chronic liver enzyme elevation; DRV/b, darunavir; DTG, dolutegravir; EFV, efavirenz; EGV/c, elvitegravir; ESLD, end-stage liver disease; HBV, hepatitis B; HCV, hepatitis C; IDU, injection drug use; INSTI, integrase strand transfer inhibitor; IQR, interquartile range; MSM, men who have sex with men; NNRTI, non-nucleoside reverse transcriptase inhibitor; NRTI, nucleoside reverse transcriptase inhibitor; PI, protease inhibitor; RAL, raltegravir; RPV, rilpivirine; TAF, tenofovir; TDF, tenofovir disoproxil fumarate.

^a^Baseline status.

### Cumulative Use of Antiretroviral Drugs and IRs of cLEE

**Figure 1.**
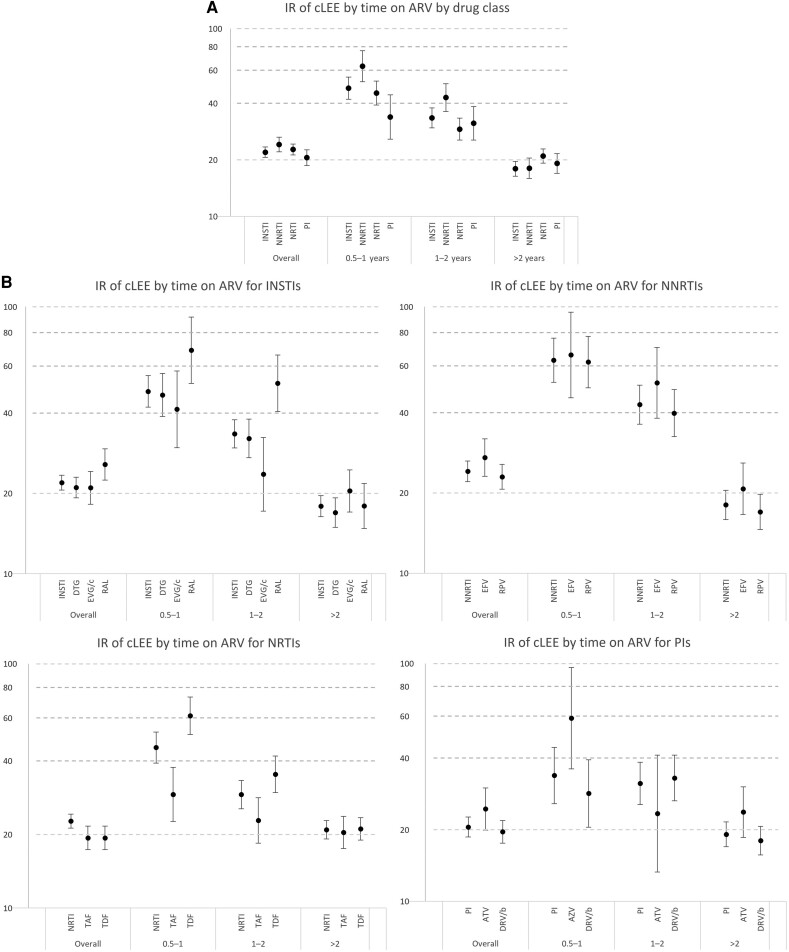
Incidence rate (95% CI) of developing cLEE after beginning a new ARV regimen to which they had not been previously exposed by time since ARV exposure and (*A*) drug class and (*B*) individual drugs. Abbreviations: ARV, antiretroviral; ATV, atazanavir (PYFU: 3763); IR, incidence rate; DRV/b, darunavir (PYFU: 16 131); DTG, dolutegravir (PYFU: 23 237); EFV, efavirenz (PYFU: 5416); EGV/c, elvitegravir (PYFU: 9053); INSTI, integrase strand transfer inhibitor (PYFU: 42 415); NNRTI, non-nucleoside reverse transcriptase inhibitor (PYFU: 20 012); NRTI, nucleoside reverse transcriptase inhibitor (PYFU: 39 625); PI, protease inhibitor (PYFU: 19 885); PYFU, person-years of follow-up; RAL, raltegravir (PYFU: 8336); RPV, rilpivirine (PYFU: 14 605); TAF, tenofovir alafenamide (PYFU: 16 011); TDF, tenofovir disoproxil fumarate (PYFU: 24 200).

There was no evidence of a cumulative association between longer ARV use and cLEE incidence for any of the considered ARVs. Overall, the cLEE IR was highest in the first 6–12 months post–new ARV initiation (IR, 45.9/1000 PYFU; 95% CI, 41.4–50.7) and declined thereafter (IR, 34.3/1000 PYFU; 95% CI, 31.4–37.4; during 1–2 years of follow-up; and IR, 18.5/1000 PYFU; 95% CI, 17.4–19.7; beyond 2 years). This was consistent across all ARV drug classes and for individual ARVs (Figure 1A and B).

### cLEE Grading Severity

Over half the individuals who developed cLEE were classified as below Grade 1 severity (>ULN–1.25×ULN—51%), followed by Grade 1 (1.25–2.5×ULN—40%), Grade 2 (2.5–5×ULN—7%), Grade 3 (5–10×ULN—2%) and Grade 4 (>10×ULN—< 1%).

### Individual ARVs Associated With cLEE

**Figure 2.**
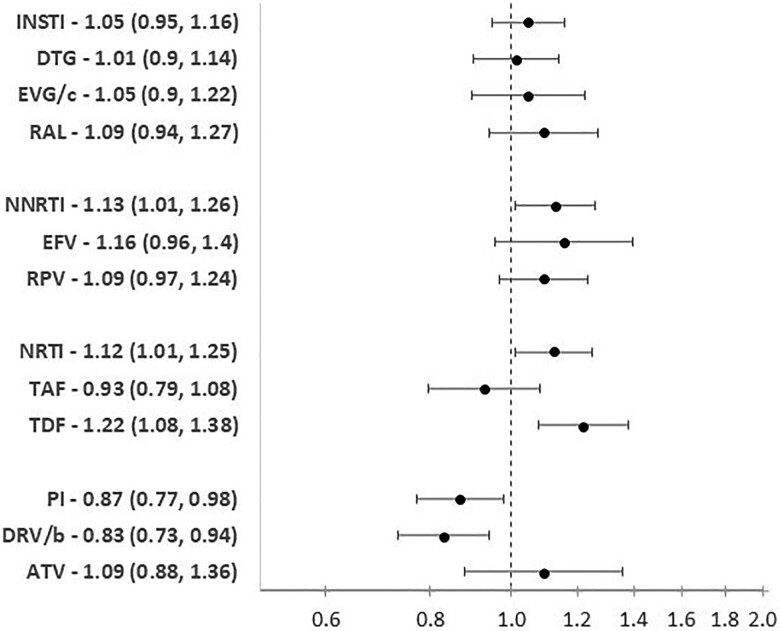
Adjusted* incidence rate ratio (95% CI) for developing chronic liver enzyme elevation after initiating a new ARV to which they had not been previously exposed by drug class and individual drug. The reference group for each model is no previous exposure to that drug. BIC did not have enough events/follow-up to analyze individually. *All models were adjusted for viral hepatitis status, nadir CD4 at baseline (<350, 350–500, ≥500 cells/mm^3^), HIV-RNA at baseline (<200, ≥200 copies/mL), region of care (Western, Southern, Northern, Eastern Europe), ARV starting year, ethnicity (White, Black, other, unknown), dyslipidemia (random total cholesterol >240 mg/dL, HDL <35 mg/dL, triglyceride >200 mg/dL, or initiation of lipid-lowering therapy), and BMI (<25, ≥25, missing). Abbreviations: ARV, antiretroviral; ATV, atazanavir; BIC, bictegravir; BMI, body mass index; DRV/b, darunavir; DTG, dolutegravir; EFV, efavirenz; EGV/c, elvitegravir; HDL, high-density lipoprotein; INSTI, integrase strand transfer inhibitor; NRTI, nucleoside reverse transcriptase inhibitor; NNRTI, non-nucleoside reverse transcriptase inhibitor; PI, protease inhibitor; RAL, raltegravir; RPV, rilpivirine; TAF, tenofovir alafenamide; TDF, tenofovir disoproxil fumarate.

ARVs associated with cLEE are shown in Figure 2. In addition to the prespecified factors of CD4 nadir, baseline HIV-RNA, and viral hepatitis status included in the model, region of care, dyslipidemia, and baseline BMI were the factors that remained statistically significantly associated with cLEE with *P* < .10, as outlined in the methods. We found no evidence of an association between use of any INSTIs and cLEE (IR, Figure 2). Use of TDF (IR, 1.24; 95% CI, 1.10–1.40) was statistically significantly associated with an increased risk cLEE, whereas use of DRV/b (IR, 0.83; 95% CI, 0.73–0.94) was associated with a decreased risk of cLEE. Overall, a higher risk of cLEE was observed for NNRTIs (IR, 1.13; 95% CI, 1.01–1.26); this was also observed for use of EFV and RPV when analyzed separately (IR, 1.27; 95% CI, 1.06–1.52; and IR, 1.18; 95% CI, 1.02–1.36; respectively), but was no longer statistically significant after adjusting for region of care (IR, 1.16; 95% CI, 0.96–1.40; and IR, 1.09; 95% CI, 0.94–1.27). Of note, the point estimates for EFV and RPV consistently remain in the same direction for the whole NNRTI class, but have fewer individuals when analyzed alone vs the combined group. A lower risk of cLEE was also observed with use of TAF (IR, 0.85; 95% CI, 0.75–0.96) but was no longer statistically significant after adjustment for region of care and TAF starting year (IR, 0.92; 95% CI, 0.79–1.08).

### Sensitivity Analyses

All individual ARV associations with cLEE were similar to the primary analyses in the sensitivity analysis using an on-treatment analysis (IR, data not shown).

**Figure 3.**
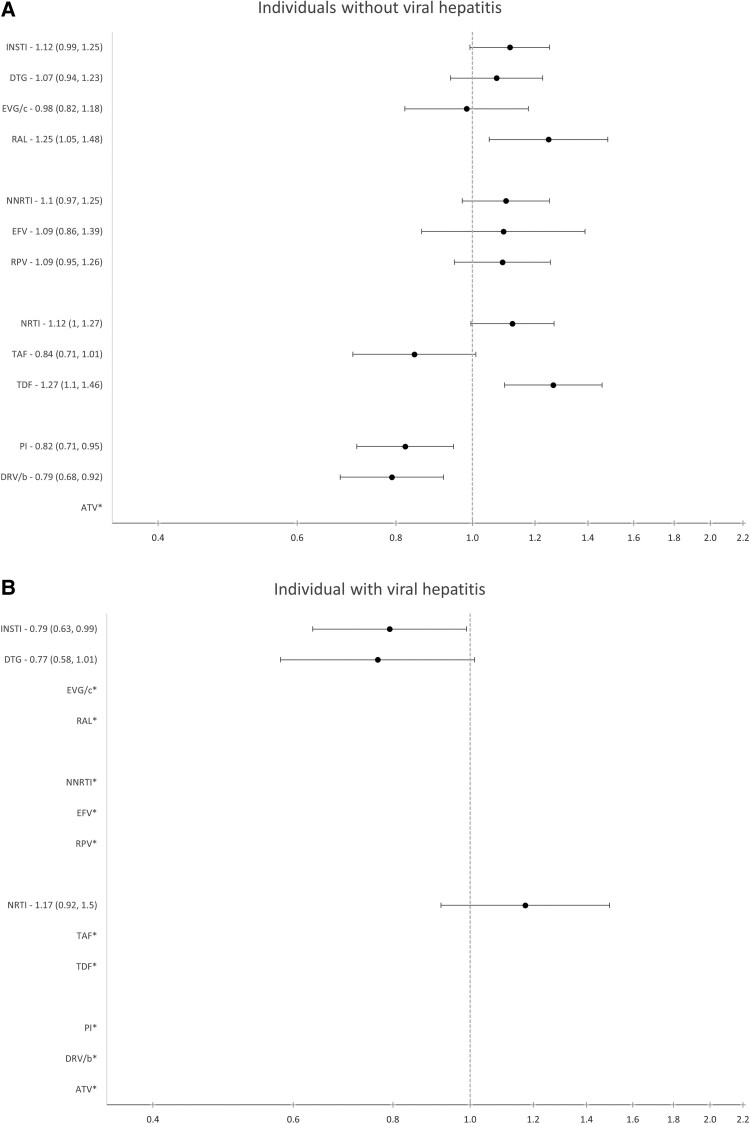
Adjusted incidence rate ratio (95% CI) for developing chronic liver enzyme elevation among those (*A*) without and (*B*) with viral hepatitis by drug class and individual drug. *There were not enough data to analyse these drugs individually. In order to have adequate power (type I and II error rates of 0.05 and 0.2, respectively) to detect an IRR of 1.4 or higher For those without hepatitis (events = 1441), we needed a follow-up ratio of 5:95, for those with hepatitis (events = 399), we needed a follow-up ratio of 23:77. The reference group for each model is no previous exposure to that drug. All models were adjusted for nadir CD4 at baseline (<350, 350–500, ≥500 cells/mm^3^), HIV-RNA at baseline (<200, ≥200 copies/mL), ARV starting year, ethnicity (White, Black, other, unknown), region of care (Western, Southern, Northern, Eastern Europe), dyslipidemia (random total cholesterol >240 mg/dL, HDL less than 35 mg/dL, triglyceride >200 mg/dL, or initiation of lipid-lowering therapy), and BMI (<25, ≥25, missing). Abbreviations: ATV, aqtazanavir; BIC, bictegravir; BMI, body mass index; DRV/b, darunavir; DTG, dolutegravir; EFV, efavirenz; EGV/c, elvitegravir; HDL, high-density lipoprotein; INSTI, integrase strand transfer inhibitor; NNRTI, non-nucleoside reverse transcriptase inhibitor; NRTI, nucleoside reverse transcriptase inhibitor; PI, protease inhibitor; RAL, raltegravir; RPV, rilpivirine; TAF, tenofovir; TDF, tenofovir disoproxil fumarate.

There was not enough power to investigate all individual ARVs among those with viral hepatitis coinfection, but we did have enough data to investigate some drug classes. Use of INSTIs was associated with a reduced risk of cLEE (IR, 0.79; 95% CI, 0.64–0.99), and there was no observed association with the other ARV drug classes (Figure 3). We also had enough power to investigate associations for individual INSTIs in those without baseline viral hepatitis and here observed an unexpected association between use of RAL and cLEE (IR, 1.25; 95% CI, 1.05–1.48). This association remained in the on-treatment analysis and when adjusting for prior AIDS and TB diagnoses. To investigate this association further, we adjusted for markers of fibrosis (IR, baseline AST to platelet ratio index [APRI], and Fibrosis-4 [FIB-4] scores), any use of other ARVs previously shown to be associated with cLEE (IR, TDF, and EFV), and stratified the analysis by starting RAL before or after 2016 (IR, as RAL utilization began to decline in 2016), but these adjustments did not remove the association among those starting RAL after 2016.

**Figure 4.**
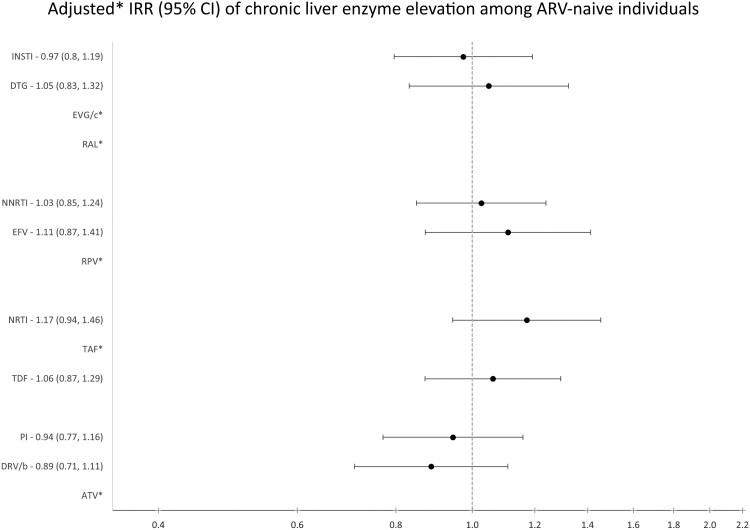
Adjusted incidence rate ratio (95% CI) for developing chronic liver enzyme elevation after initiating a new ARV to which they had not been previously exposed among those who were ART-naïve before baseline by drug class and individual drug using the RESPOND data set. *There were not enough data to analyze these drugs individually. In order to have adequate power (type I and II error rates of 0.05 and 0.2, respectively) to detect an IRR of ≥1.4 for those who were ART-naïve before baseline (events = 599), we needed a follow-up ratio of 14:86. The reference group for each model is no previous exposure to that drug. All models were adjusted for viral hepatitis status, nadir CD4 at baseline (<350, 350–500, ≥500 cells/mm^3^), HIV-RNA at baseline (<200, ≥200 copies/mL), ARV starting year, region of care (Western, Southern, Northern, Eastern Europe), ethnicity (Black, White, other, unknown), dyslipidemia (random total cholesterol >240 mg/dL, HDL <35 mg/dL, triglyceride >200 mg/dL, or initiation of lipid-lowering therapy), and BMI (<25, ≥25, missing). Abbreviations: ATV, aqtazanavir; BIC, bictegravir; BMI, body mass index; DRV/b, darunavir; DTG, dolutegravir; EFV, efavirenz; EGV/c, elvitegravir; HDL, high-density lipoprotein; INSTI, integrase strand transfer inhibitor; NNRTI, non-nucleoside reverse transcriptase inhibitor; NRTI, nucleoside reverse transcriptase inhibitor; PI, protease inhibitor; RAL, raltegravir; RPV, rilpivirine; TAF, tenofovir; TDF, tenofovir disoproxil fumarate.

Among those who were ART-naïve before baseline, trends were similar to the main analysis, but with significantly lower statistical power (Figure 4).

We investigated those who initiated only 1 of EFV, RPV or TDF. The association with TDF/RPV and cLEE remained (IRR, 1.22; 95% CI, 1.04–1.41; and IRR, 1.06; 95% CI, 0.82–1.38), and there was no longer an association with EFV and cLEE, although the numbers were greatly reduced (IRR, 0.98; 95% CI, 0.63–1.52). Among those who initiated DRV/b without TDF, we too saw results similar to those of the main analysis (IRR, 0.93; 95% CI, 0.76–1.14), although the sample size was significantly smaller.

### Other Factors Associated With cLEE

Besides ARV exposure, as expected we found other factors to be associated with cLEE. Table 2 shows these factors modeled without individual ARV exposure, but these results were consistent across all our models. We found ethnic origin (IRR, 0.67; 95% CI, 0.58–0.78; Black vs White), geographical region of care (IRR, 0.86; 95% CI, 0.76–0.98; IRR, 1.09; 95% CI, 0.93–1.27; IRR, 1.45; 95% CI, 1.26–1.68; for Southern, Northern, Eastern, vs Western Europe), ARV starting year (IRR, 0.88; 95% CI, 0.89–0.97; ≥2016 vs <2016), viral hepatitis status (IRR, 1.30; 95% CI, 1.16–1.45; positive vs negative), dyslipidemia (IRR, 1.33; 95% CI, 1.21–1.45), and BMI (IRR, 1.46; 95% CI, 1.32–1.62; for ≥25 compared with <25) to be associated with cLEE (Table 2).

**Table 2.** Other Factors Associated With Developing Chronic Liver Enzyme Elevation in a Multivariable Model After Initiating a New ARV to Which They Had Not Been Previously Exposed Using the RESPOND Data Set

		IRR*	95% CI		*P* Value
Ethnicity	White	1.00	…	…	
…	Black	0.67	0.58	0.78	<.001
…	Other	1.50	1.24	1.81	<.001
…	Unknown	0.94	0.79	1.11	.45
Geographical region of care	Western Europe	1.00	…	…	
…	Southern Europe	0.86	0.76	0.98	.026
…	Northern Europe	1.09	0.93	1.27	.313
…	Eastern Europe	1.45	1.26	1.68	<.001
ARV starting year*	<2016	1.00	…	…	
…	≥2016	0.88	0.80	0.97	.012
Baseline CD4 nadir, cells/mm³	<350	1.00	…	…	
…	350–500	1.02	0.89	1.16	.79
…	>500	1.02	0.88	1.19	.779
Baseline HIV-RNA, copies/mL	<200	1.00	…	…	
…	≥200	1.05	0.95	1.17	.312
Hepatitis status	Negative	1.00	…	…	
…	Positive	1.30	1.16	1.45	<.001
…	Unknown	1.35	1.09	1.67	.006
Dyslipidemia	…	1.33	1.21	1.45	<.001
Baseline BMI*	<25	…	…	…	
…	≥25	1.46	1.32	1.62	<.001
…	Missing	1.14	1.01	1.29	.035

*Abbreviations: ARV, antiretroviral; BMI, body mass index; IRR, incidence rate ratio; RESPOND, International Cohort Consortium of Infectious Diseases.

These results are from a multivariable model that does not adjust for individual ARV exposures. CD4 nadir, HIV-RNA, and hepatitis status were confounders that we determined a priori would be included in all models. The other remaining factors were consistently associated with cLEE in all models (data not shown). Age, sex, HIV transmission risk group, and smoking status were nonsignificant in the multivariable models and were not included as covariates.

## DISCUSSION

This is the largest international study to systematically examine the relationship between use of commonly used ARVs, including individual INSTIs and cLEE, over the last decade. We found that cLEE is common among contemporarily treated individuals, although most instances were low grade and more frequent in the first year after initiating a new ARV. With a median follow-up in excess of 5 years, we found no short-term risk of ALT elevation with the use of INSTIs, even after adjusting for confounders including viral hepatitis, BMI, CD4 cell counts, HIV-RNA, dyslipidemia, and diabetes mellitus. We confirmed previous studies’ findings that use of EFV, RPV, and TDF was associated with an increased risk of cLEE [22, 24, 28, 29, 30]; although EFV and RPV did not reach statistical significance after adjusting for region of care, the point estimate remained in the same direction, just losing statistical significance. Additionally, use of TAF and DRV/b was associated with lower risk of cLEE, although TAF did not reach statistical significance after adjusting for region of care and calendar year.

From the outset, clinical trials for INSTIs suggest no concern for hepatotoxicity compared with other drugs [31–33, 34], although liver enzyme elevations can affect 1%–10% of individuals [29, 35–40]. It is important to monitor the liver safety of drugs over a longer term and under real-life conditions in a heterogenous population beyond what clinical trials allow. Two previous national studies found use of INSTIs to be associated with a lower risk of liver enzyme elevations in the United States and Italy, respectively [21, 41]. Of note, the comparison groups were different in these prior studies of INSTI use with liver outcomes, and liver enzyme elevation was defined differently than in our analysis. Wood et al. showed a lower risk of cLEE (defined as ALT ≥1.25 times the ULN on at least 2 visits, for a duration of ≥6 months within 2 years) in individuals receiving INSTIs compared with those who received boosted PIs, and Taramasso et al. showed lower liver enzyme elevation (ALT >2.5 times the ULN in individuals with normal baseline levels, or 2.5 times baseline value for those with elevated baseline ALT) in INSTIs compared with NNRTIs. This contrasts with our analysis, where we compared INSTIs with all other drugs. With a median follow-up of >4 years, this supports previous work from smaller cohorts suggesting that INSTI-based regimens do not lead to elevated liver enzymes.

Although we observed no association of INSTIs with cLEE compared with any other drug in the main analysis, we surprisingly observed a seemingly protective effect of INSTIs in the group with viral hepatitis at baseline. It should be noted that individuals with baseline viral hepatitis still had to meet our inclusion criteria of a normal ALT at baseline, so these individuals were highly selected and not representative of the general hepatitis-positive population. We did attempt to investigate those with chronic hepatitis C, including only those with a positive HCV-RNA measurement at baseline, but there were too few individuals to analyze (n = 434).

We did observe an increased risk of cLEE with use of RAL among those without viral hepatitis B or C. Of note, as previously reported in RESPOND, RAL utilization declined substantially over time [42]; before 2016, 13% of the cohort initiated RAL compared with only 3% after 2016. To investigate this positive association further, we stratified the analysis by year of starting RAL (<2016 and ≥2016) and additionally by TB diagnosis, AIDS diagnosis, and CVD. The association between cLEE and RAL was not present before 2016 when RAL was most used, and the association with cLEE was strongest among those starting in later years, when very few individuals initiated RAL. We therefore believe this finding is due to unmeasured confounding as RESPOND does not capture other clinical reasons that might be linked to liver injury (eg, other drugs with hepatotoxic potential) for individuals starting RAL in later years, when other ARVs were preferred.

The increased risk of cLEE associated with NNRTIs as a class and TDF utilization builds on evidence from previous studies. Some individuals are treated with these drugs concomitantly; to show that the effects we observed from each drug were independent, we performed a sensitivity analysis investigating only individuals prescribed 1 of these drugs. The magnitude of our effects remained similar to the main results for TDF and RPV, although the sample size was greatly reduced. The association between EFV and cLEE was removed when we analyzed individuals not concomitantly taking TDF or RPV (IRR, 0.96; 95% CI, 0.61–1.52). This could be due to the association being driven by TDF or the decreased sample size of this analysis (n = 17 106 and 1932 cLEE events vs n = 8533 and 754 events).

NNRTIs were associated with an increase in cLEE, driven by EFV and RPV utilization (Figure 2). Given that the point estimates for EFV and RPV are consistently in the same direction as NNRTIs as a class, we believe that EFV and RPV are the main drivers of the NNRTI class association as they are the most commonly used NNRTIs. The numbers of individuals starting EFV (n = 882) and RPV (n = 2623) are substantially smaller than NNRTIs as a class (n = 3504), so the lack of statistical significance is likely due to lack of power. Previous data have shown that acute ALT elevations >5 times the ULN occur in 1%–8% of individuals on EFV [28, 29, 30], and this rate is higher among those who are coinfected with HCV [29, 43]. We have shown that in addition to acute liver injury, EFV also contributes to longer-term liver enzyme elevations, and there is a similar risk for cLEE among those mono-infected with HIV and those also coinfected with HCV and/or HBV, although this was not statistically significant due to the limited number of individuals included in this analysis.

Previous observational data from the D:A:D and Swiss HIV Cohort Studies have also shown an association between TDF and elevated ALT levels in those with and without viral hepatitis coinfection [22, 24], consistent with our findings. We found a trend for slightly reduced risk of cLEE with TAF, although this did not reach statistical significance. This is supported by data from the Swiss HIV Cohort Study and the Canadian Hepatitis B Network showing that among those with elevated ALT, switching from TDF to TAF significantly reduces ALT elevations [22, 44]. It is worth noting that in RESPOND there is likely confounding by indication with TAF utilization given that it has a more favorable safety profile compared with TDF.

Importantly, our data add to the evidence showing that there are long-term chronic ALT elevations that should be monitored with NNRTIs and TDF.

We found no association between cLEE and PIs, which is supported by other recent findings [45–48]. However, we did observe a decreased risk of cLEE associated with DRV/b use, which has not been shown previously. DRV/b is often used in individuals challenged by adherence and/or drug resistance due to its high genetic barrier to HIV resistance [49]. It is therefore encouraging that hepatotoxicity does not appear to be a concern with DRV/b use, regardless of viral hepatitis status, although confounding by indication might play a role in our findings. DRV/b is also often concomitantly prescribed with TDF, which is associated with cLEE. Among those prescribed DRV/b without TDF, we found results similar to those of the main analysis, although the numbers were greatly reduced.

Most (92%) of the individuals in our cohort defined as having cLEE were not classified as having the ALT elevation component of the drug-induced liver injury definition (DILI—ALT > 2 ULN) [50], so for most individuals the clinical relevance of cLEE is not yet clear. Previous research has shown mixed effects of elevated liver enzymes on all-cause mortality [45, 51, 52], but the definitions used for ALT elevations were not consistent among these studies. Our data do not yet allow for further investigation of cLEE and mortality, as the median time from cLEE onset to the end of follow-up (IQR) was 2.8 (1.3–4.5) years.

### Limitations and Strengths

This study has some limitations. As with all observational studies, the validity of our estimates relies on the untestable assumption that we have appropriately adjusted for unmeasured confounding. There are likely unmeasured factors and/or unknown confounders that predict cLEE in this population (eg, drug–drug interactions or drugs with unfavorable hepatic safety profiles). Although it is not possible to know the magnitude of these unmeasured factors, we have adjusted for the most commonly measured factors known to be associated with cLEE including viral hepatitis status, BMI, CD4 cell counts, HIV-RNA, dyslipidemia (including use of lipid-lowering drugs), and diabetes mellitus. We do not believe that there are other major factors that would confound the relationship between ARV and cLEE. Alcohol use data were not systematically collected in our cohort at the time, and we were unable to adjust for this. It is unlikely that alcohol confounds the relationship between cLEE and ARV that we observed, as alcohol is unlikely to have a large impact on a physician's ARV selection for an individual with normal liver function. While we acknowledge that people with HIV are prescribed ARVs in combinations, due to the large number of different combinations available it is not feasible to analyze all unique regimens. Further, focusing on regimens rather than individual ARVs would have made it challenging to disentangle which of the ARVs in the combination drives the association seen. We have also included individuals previously exposed to ARVs, which could impact liver function, but an inclusion criterion of this analysis was having normal ALT at baseline, and in sensitivity analyses models were further adjusted for ARVs previously associated with liver toxicity and for APRI and FIB-4. We have also tried to account for this by limiting the analysis to ART-naïve individuals before baseline, and among the drugs with enough power to analyze, we found similar results as in the main analysis. Additionally, we did not have adequate data on all other potentially hepatotoxic drugs prescribed in conjunction with ARVs, so we cannot rule out drug–drug interactions playing a role in our findings. We did, however, adjust for those with prior AIDS or TB diagnoses as these individuals might be prescribed RAL preferentially over other ARVs because RAL does not have the same potential for interactions with TB drugs. The median age of our cohort (IQR) was 47 (38–55) years, 26% of our cohort was age 50–60 years, and 14% was older than 60. It is possible that there would be a higher incidence of comorbidities, drug–drug interactions, and liver injuries among older individuals, but we did not see an association between older age and increased risk of cLEE. We were also unable to investigate all newer ARVs including doravirine and cabotegravir due to the limited number of individuals initiating these drugs. We could not fully distinguish those with active chronic HCV and HBV infections from those with inactive or cleared infections due to data quality, and those with viral hepatitis in our study would have had normal ALT at baseline as per our inclusion criteria. Finally, our data are from clinical cohorts based in Europe and Australia, and the results might not be generalizable beyond this setting.

There are many strengths of this study, notably its large size and long-term and recent follow-up with established cohorts throughout Europe and Australia. We therefore were able to adequately investigate newer ARVs including individual INSTIs that have not been investigated for chronic liver injury previously on a large scale in this setting. RESPOND is in a unique position to investigate this due to the sample size and heterogenous clinical settings where there is enough variability in ARV prescription combinations to investigate drugs individually.

In conclusion, we have analyzed a large observational study and systematically examined the relationship between commonly used ARVs and chronic liver injury, assessed using liver enzymes, and found no short-term safety concerns with the use of INSTIs. We did find an elevated risk of cLEE with use of NNRTIs (driven by EFV, RPV) and TDF, a decreased risk of cLEE with DRV/b use, and a nonsignificant trend toward decreased risk of cLEE with TAF. Further research is needed to monitor longer-term associations with cLEE, particularly for INSTIs, associations with other liver end points including markers of fibrosis, and the impact of cLEE on mortality for newer ARVs in different settings.

## Supplementary Material

ofae308_Supplementary_Data

## Supplementary Data

Supplementary materials are available at *Open Forum Infectious Diseases* online. Consisting of data provided by the authors to benefit the reader, the posted materials are not copyedited and are the sole responsibility of the authors, so questions or comments should be addressed to the corresponding author.
