# Supplementary material for: Chronic Liver Enzyme Elevation and Use of Contemporary ARVs Among People With HIV
Source: Open Forum Infect Dis. 2024 Jun 8;11(6):ofae308. doi: 10.1093/ofid/ofae308 (PMC11196901; doi:10.1093/ofid/ofae308)
Supplement: ofae308_Supplementary_Data [file ofae308_supplementary_data.docx]

Supplementary Table 1: Baseline characteristic of those included in the analysis compared to the whole RESPOND cohort.

|  |  | **Overall RESPOND database (n=39267)** | | **Individuals included in the analysis (n=17106)** | |
| --- | --- | --- | --- | --- | --- |
|  |  | **N** | **%** | **N** | **%** |
| **Gender** | **male** | 29524 | 75.20 | 13018 | 76.10 |
|  | **female** | 9667 | 24.60 | 4046 | 23.70 |
|  | **other/unknown** | 76 | 0.20 | 42 | 0.20 |
| **Ethnic Origin** | **White** | 27342 | 69.60 | 12090 | 70.70 |
|  | **Black** | 5412 | 13.80 | 2390 | 14.00 |
|  | **Other** | 2102 | 5.40 | 780 | 4.60 |
|  | **Unknown** | 4411 | 11.20 | 1846 | 10.80 |
| **HIV risk** | **MSM** | 18346 | 46.70 | 8346 | 48.80 |
|  | **IDU** | 4951 | 12.60 | 1686 | 9.90 |
|  | **Heterosexual** | 13326 | 33.90 | 6019 | 35.20 |
|  | **Other** | 971 | 2.50 | 395 | 2.30 |
|  | **Unknown** | 1673 | 4.30 | 660 | 3.90 |
| **Geographical Region** | **Western Europe** | 17480 | 44.50 | 9534 | 55.70 |
|  | **Southern Europe** | 7355 | 18.70 | 3664 | 21.40 |
|  | **Northern Europe** | 9202 | 23.40 | 1969 | 11.50 |
|  | **Eastern Europe** | 5230 | 13.30 | 1939 | 11.30 |

Supplementary Figure 1: Flow chart showing the selection of patients in RESPOND used in the analysis.

39,267 individuals enrolled in RESPOND

5,052 individuals came from cohorts without 70% ALT data completeness

7,217 did not begin a new ARV regimen after 2012

1,521 were missing an HIV-RNA or CD4 cell count in the year preceding baseline

6,140 had an abnormal ALT in the year preceding baseline

17,106 individuals were included in the analysis

1,891 were missing an ALT measurement in the year preceding baseline

340 did not have an ALT taken after baseline
